# *Alu* SINE analyses of 3,000-year-old human skeletal remains: a pilot study

**DOI:** 10.1186/s13100-016-0063-y

**Published:** 2016-04-18

**Authors:** Maximilian Kothe, Verena Seidenberg, Susanne Hummel, Oliver Piskurek

**Affiliations:** Department of Historical Anthropology and Human Ecology, Georg-August-University, 37073 Göttingen, Germany

**Keywords:** Transposable elements, *Alu*, SINE, retrotransposon, aDNA, *ancient*DNA, DNA preservation, Lichtenstein cave, Bronze Age

## Abstract

**Background:**

As Short Interspersed Elements (SINEs), human-specific *Alu* elements can be used for population genetic studies. Very recent inserts are polymorphic within and between human populations. In a sample of 30 elements originating from three different *Alu* subfamilies, we investigated whether they are preserved in prehistorical skeletal human remains from the Bronze Age Lichtenstein cave in Lower Saxony, Germany. In the present study, we examined a prehistoric triad of father, mother and daughter.

**Results:**

For 26 of the 30 *Alu* loci investigated, definite results were obtained. We were able to demonstrate that presence/absence analyses of *Alu* elements can be conducted on individuals who lived 3,000 years ago. The preservation of the ancient DNA (aDNA) is good enough in two out of three ancient individuals to routinely allow the amplification of 500 bp fragments. The third individual revealed less well-preserved DNA, which results in allelic dropout or complete amplification failures. We here present an alternative molecular approach to deal with these degradation phenomena by using internal *Alu* subfamily specific primers producing short fragments of approximately 150 bp.

**Conclusions:**

Our data clearly show the possibility of presence/absence analyses of *Alu* elements in individuals from the Lichtenstein cave. Thus, we demonstrate that our method is reliably applicable for aDNA samples with good or moderate DNA preservation. This method will be very useful for further investigations with more *Alu* loci and larger datasets. Human population genetic studies and other large-scale investigations would provide insight into *Alu* SINE-based microevolutionary processes in humans during the last few thousand years and help us comprehend the evolutionary dynamics of our genome.

**Electronic supplementary material:**

The online version of this article (doi:10.1186/s13100-016-0063-y) contains supplementary material, which is available to authorized users.

## Background

After the discoveries of Barbara McClintock on *Zea Mays* [1, 2], much research has been conducted in the field of transposable elements (TEs). It is now known that TEs, long classified as junk DNA [3], have major effects on the genomes of all organisms. For example, they may affect gene functions or alternate transcription rates [4–9]. In eukaryotes, TEs are mostly inherited vertically from generation to generation and in rare cases horizontally, e.g. via a viral vector [10]. In humans, TEs make up a great part of the total genome. Estimates vary from ~45 % [11] to ~69 % [12]. Thousands of new TE loci have recently been identified in the human 1,000 Genome Project [13, 14]. The transposition mechanism of TEs can generally be divided into two classes: class I retrotransposons and class II DNA transposons. While DNA transposons move via a “cut-and-paste” mechanism, the retrotransposons move by a “copy-and-paste” mechanism. As class l elements, the non-autonomous **S**hort **In**terspersed **E**lements (SINEs) present the largest group of TEs in eukaryotic genomes in terms of copy numbers [11]. More than 200 SINE families have been identified so far [15]. Their sequence information can be retrieved at SINEBase [15] and RepBase [16]. The retrotransposition mechanism of a SINE requires a **L**ong **In**terspersed **E**lement (LINE)-encoded protein from a LINE partner with reverse transcriptase and endonuclease activity [17].

The absence of an element at a specific locus can be described as the ancestral state, whereas presence is the derived state [18, 19]. Due to the irreversibility of an insertion and its homoplasy-free character, SINE insertions are a powerful tool for phylogenetic analyses [20, 21]. The most abundant SINEs in humans are the primate-specific *Alu* elements, reaching a copy number of about 1.1 million [11, 22]. Their partner LINEs are L1 elements that represent a family of mammalian retrotransposons that have been replicating and evolving for more than 100 Myr [23]. *Alu* elements usually have a length of approximately 300 base pairs. They began to expand with the primate radiation 65 Mya and peaked in activity 40 Mya. It is believed that only a few “Master Genes” are retropositionally competent [24]. Due to accumulations of new mutations, over evolutionary time, new *Alu* subfamilies are created. The 7SL RNA-derived *Alu* elements can be classified in three subfamilies J, S and Y, with *Alu*J being the oldest, followed by *Alu*S and *Alu*Y as the youngest and only active subfamily [22]. Within the *Alu*Y elements, the subfamilies *Alu*Ya5 and *Alu*Yb8 are the groups with the largest numbers of copies. Some of these elements retrotransposed so recently that they are absent in other primate lineages and are even polymorphic between and within human populations [25–28]. These polymorphic elements are perfectly suited for population genetic and phylogenetic studies. In cases of rapid radiation of taxa or simultaneous lineage divergence, some TEs may not show the real phylogenetic state. This phenomenon is called incomplete lineage sorting [29–32]. Nonetheless, polymorphic *Alu* elements are excellent ancestry markers for resolving relationships within and between human populations [33]. In a genome-wide study of polymorphic TEs in 2,504 individuals across 26 human populations, Rishishwar et al. [14] recently showed that the genetic diversity represented by TE polymorphisms, mainly by *Alu* elements, reflects known patterns of human evolution. *Alu* elements and TEs in general insert nearly randomly into the genome, exist in large copy numbers, and are mostly non-autonomous [34, 35]. Our genome is constantly in evolutionary change [36]. Normally, the long-term effects of gene evolution and function alternation become visible [37, 38]. The effects of short-term or microevolutionary processes can be detected by analyzing the presence/absence situations of human specific *Alu* elements.

For such analyses, human remains with well-preserved DNA are required. Usually, DNA degradation in bones is too advanced for analyses of fragments that exceed 200–300 base pairs [39, 40], but it was proven, for example, that larger fragments of 397 bp from bone samples of the Lichtenstein cave can be amplified, too [41]. The main causes of DNA loss in remains are autolysis directly after death, hydrolisis, and oxidation [42, 43]. The degree of post-mortem DNA degradation depends on environmental factors such as acid conditions, microbial activity, and high mean temperatures [42]. On the other hand, constant low temperatures and neutral or slightly alkaline pH-values provide optimal conditions for DNA preservation [40, 42]. These conditions are found in the Lichtenstein cave near Osterode in Lower Saxony, Germany. For thousands of years, the cave has had a constant temperature of 6–8 °C. Additionally, the skeletal remains were coated with a gypsum layer, which causes a slightly basic environment and is thus perfect conditions for preserving bones and DNA. Previous studies on these remains revealed kinship relations between many individuals [44, 45]. These results are based on genetic fingerprinting, mtDNA, and Y-haplotypes [46, 47]. In the present work, a triad of father, mother, and daughter [44, 45] was chosen for the investigations. Besides kinship calculations, STR fingerprints are used for personal identification due to the unique pattern of STRs. In this study, a genetic STR fingerprint multiplex analysis is used to ensure the authenticity of DNA extracts by monitoring for potential contaminations from the laboratory staff.

Considering the rules of Mendelian inheritance, the known kinship relation between the chosen individuals is helpful for revealing potential false negative results. Especially in aDNA analyses, the phenomenon of allelic dropout is common. Large alleles are more frequently affected by allelic dropout than short alleles are, depending on the degree of DNA fragmentation of the remains [39].

In the present work, the presence/absence situation of 30 *Alu* loci was investigated for three members of a prehistoric family (father, left femur DO 1911; mother, left femur DO 3756; daughter, left femur DO 3750) and two modern individuals of Caucasian origin as positive controls. A presence band is defined as the *Alu* locus where the element is inserted, resulting in a long amplification fragment, an absence band as the locus where the *Alu* element is not inserted, which appears as a shorter fragment on the gel. We show that it is possible to amplify, *Alu* loci, including flanking regions with fragment lengths up to 500 bp, for the 3,000-year-old remains in the Lichtenstein cave. We also demonstrate an alternative approach for cases in which, due to DNA degradation, the classic PCR approach failed to amplify the longer presence fragments. Additionally, we give a brief statement about questions to be raised in further investigations.

## Results/Discussion

### Presence/Absence analyses

The study illustrates the presence or absence of 30 *Alu* loci in three prehistoric and two modern individuals. The precise genomic locations of all 30 loci are listed in the additional files (Additional file 1). The positions are based on the human genome assembly GRCh38.p5 (see online database ensembl.org) [48]. The results of the classical PCR approach and internal *Alu* amplification are presented in Table 1 (for the molecular approach, see methods). In addition to the three prehistoric samples, two modern positive controls were investigated (CAU_1 and CAU_2). CAU_1 originates from a Caucasian American person; CAU_2 is a person of Central European origin. Randomly selected loci were chosen and verified by cloning and sequencing (accession numbers KU323383-KU323387) to ensure the authenticity of the bands (Additional file 2).

**Table 1:**
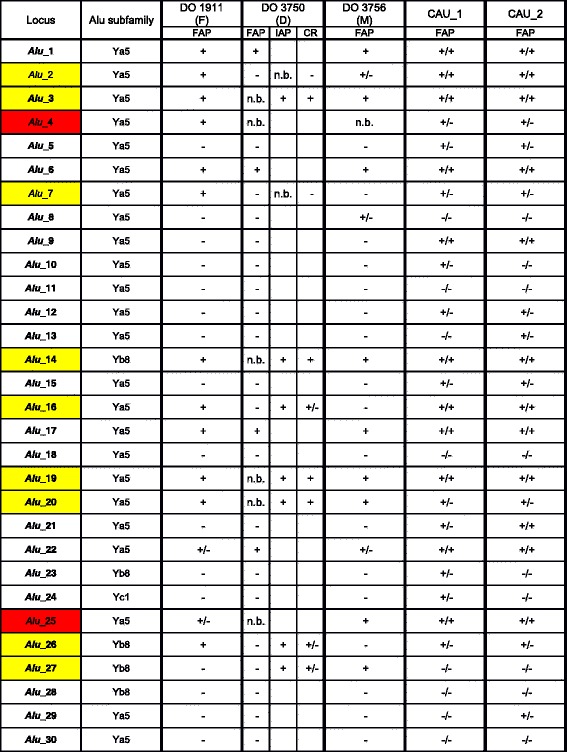
*Alu* presence/absence results for all examined individuals

-; -/-: genomic sequence without *Alu* insert

+; +/+: genomic sequence including *Alu* insert

n.b.: no band

FAP: result after amplification with flanking *Alu* primers

Yellow boxes: amplification with internal *Alu* primer (IAP)

CR: Combined result of both approaches for DO 3750

Bold: complete result

Red boxes: not further investigated (see Results/Discussion)

The homozygous results for the prehistoric individuals are represented only by “+” or “‐”, to include the possibility of allelic dropout events. In contrast, the homozygous results for the modern samples are indicated by “+/+” or “-/-”, due to the reliability of analyses of modern DNA. In this study, a “definite result” is defined as successful product amplification for all three samples (father, mother and daughter) per *Alu* locus based on both molecular approaches (FAP and if necessary IAP, see Table 1) upon condition that the family situation is congruent. *Alu* loci that are marked yellow show the incongruence of the family situation in relation to Mendelian inheritance, or the amplification failed completely. This is best explained by the phenomenon of allelic dropout, which is known and common in aDNA analyses. The presence band is periodically not amplified because allelic dropout usually impacts larger alleles. A low number of intact targets is one reason why some alleles may not occur at all or may not reach the detection limits of the devices of electrophoresis [39].

**Fig. 1:**
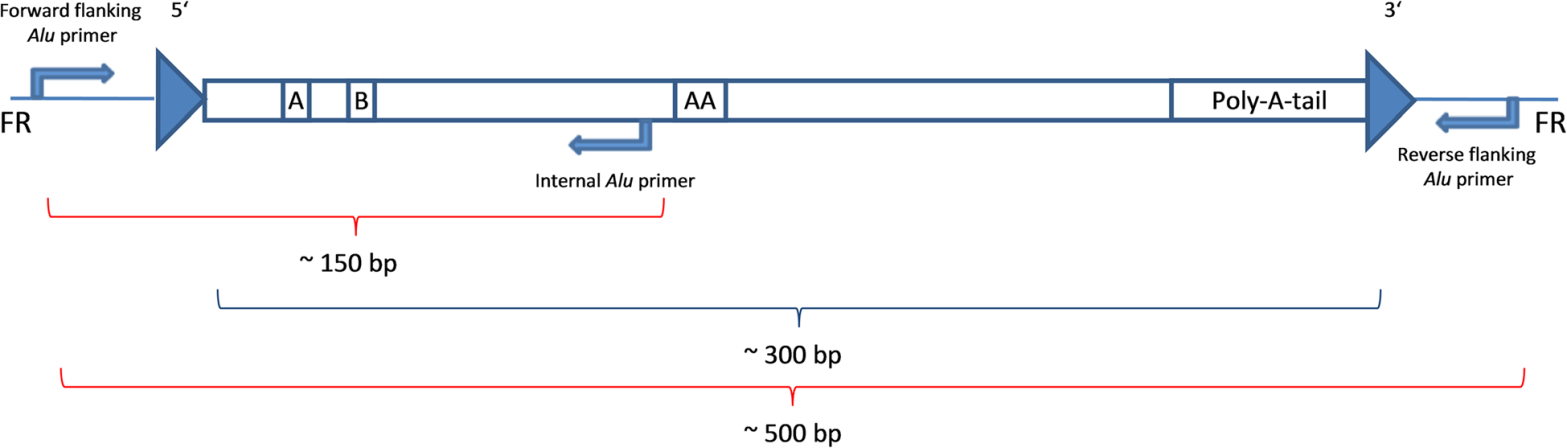
The amplification via an internal *Alu* primer results in amplicons of ~150 bp. The reverse flanking *Alu* primer is replaced by an internal *Alu* subfamily specific primer. The internal primer is located at the 3′ sequence of the left *Alu* monomer prior to the A-rich region in the middle of the element. The amplification via flanking *Alu* primers results in amplicons of ~500 bp. The large arrows at the 5′ and 3’ ends indicate the target site duplications

**Fig. 2:**
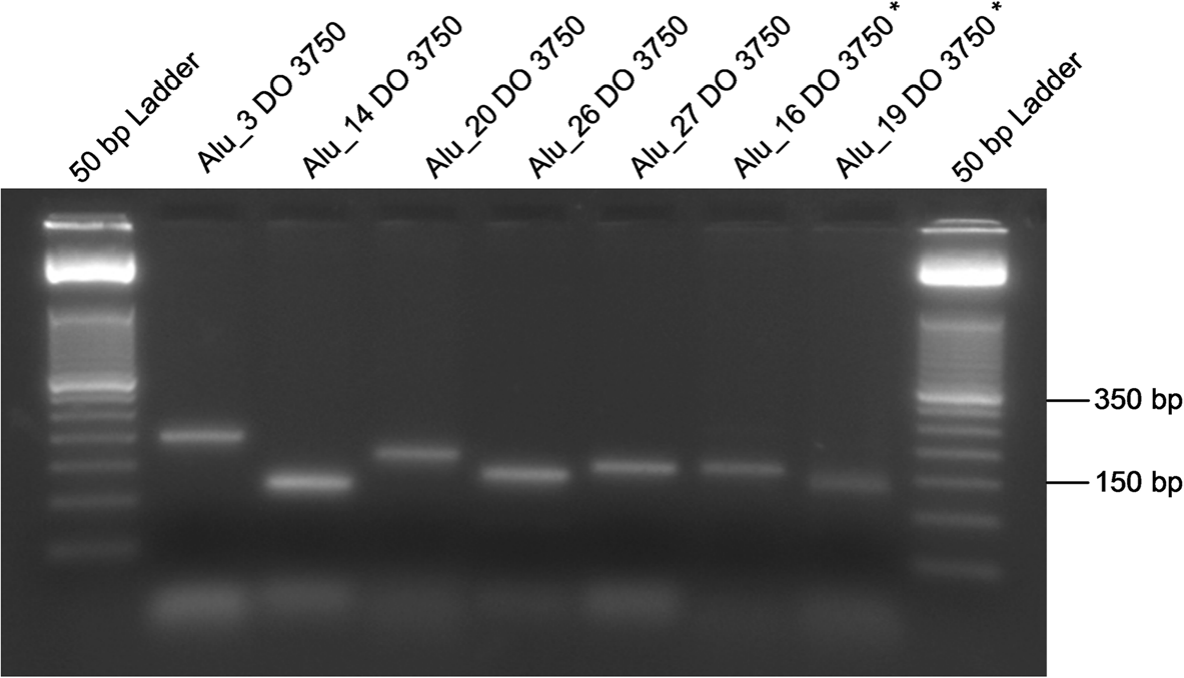
The photo shows seven successfully amplified amplicons of an internal *Alu* primer-based amplification. The expected fragment lengths vary from 118-194 bp. The marks on the base pair ladder are situated at 150 bp and 350 bp. For these seven *Alu* loci, the presence band for DO 3750 was proved via internal *Alu* amplification. The asterisks indicate reverse *Alu* inserts. In these cases, the primer pairings are an internal *Alu* primer with the reverse *Alu* flanking primer, whereas the samples without asterisk were amplified with an internal *Alu* primer and the forward *Alu* flanking primer

Obviously incomplete and incongruent results were subjected to an alternative molecular approach. Using an internal *Alu* primer, the predicted fragment length of the amplicon was reduced to ~150 bp (Fig. 1). The internal primers were designed based on an alignment of *Alu* sequences of the respective subfamily and consequently are very specific to each *Alu*Y subfamily as described by Nelson et al. [49] or Kass and Batzer [50]. This type of amplification worked in seven cases for the sample DO 3750 (Fig. 2). The heterozygous results for *Alu*_16, *Alu*_26 and *Alu*_27 for the daughter (‘CR’ in Table 1) represent a combination of both amplification approaches. Further internal *Alu* primer analyses were not possible, due to a depleted DNA extract (*Alu*_4, *Alu*_25; marked red). Loci with exclusively absence bands for the prehistoric individuals, in particular, should be checked by internal *Alu* amplification. The advantage of this method is that the amplification of short fragments (usually ~150 bp) still proves the presence of an insert. In this study, this approach was applied only in those cases where the *Alu* amplification results are not in concordance with the family situation or where the amplification totally failed for DO 3750. Based on previous analyses on this prehistoric triad, it is known that the DNA is less well-preserved in DO 3750 and best-preserved in DO 1911. Consequently, the chance of allelic dropout events for DO 3750 is more likely than for DO 3756 and DO 1911. Fragments of such short lengths (~150 bp) are usually not affected by allelic dropout. However, the internal primer approach cannot be applied in isolation because it does not indicate heterozygous states.

### Proof of kinship in the prehistoric samples and authenticity

The authenticity of the aDNA was ensured by amplifying STR-based genetic fingerprints. Table 2 presents the consensus results of the Heptaplex STR analyses for the DNA extracts used. Table 3 shows the consensus results for seven additional STR systems. A full list of all results achieved can be found in the supplementary files (Additional file 3). Nearly all amplifications were conducted with DNA material taken from the left femur. The genetic fingerprint results of the STR systems D16S539, D2S1338 and D19S433 for the daughter were not achieved with DNA material from her left femur (DO 3750), but from her left humerus (DO 3994). The results of all 13 STR systems for the three prehistoric individuals were used for a kinship calculation resulting in a kinship probability of 99.999 %. All single allele frequencies were taken from the online database allstr.de [51]. Given this proven kinship, false homozygous *Alu* results for the daughter can be clearly detected by contradiction between the parental alleles and the Mendel’s laws of inheritance.

**Table 2:** Heptaplex based fingerprint results for all investigated individuals

	Individual	Amelo	D13S317	D21S11	D18S51	TH01	D5S818	FGA
prehistoric	DO1911 (F)	X/Y	12/12	30.2/32.2	15/17	9.3/9.3	11/12	21/22
	DO3756 (M)	X/X	8/9	28/29	16/16	9/9.3	12/12	21/23
	DO3750 (D)	X/X	9/12	29/32.2	16/17	9/9.3	11/12	21/23
modern	Cau_1	X/Y	12/13	28/32.2	15/18	9/9.3	11/13	20/21
	Cau_2	X/Y	10/11	28/31.2	14/14	9.3/9.3	8/12	22/24

**Table 3:** Additional fingerprint results for the prehistorical individuals

Individual	D3S1358	VWA	D8S1179	D7S820	D16S639	D2S1338	D19S433
DO 1911 (F)	16/18	17/19	13/13	8/11	12/13	18/19	14/15
DO 3756 (M)	18/18	17/19	12/15	10/10	12/13	24/25	13/14
DO 3750 (D)	16/18	17/17	12/13	8/10	12/13*	19/25*	14/14*

*: The data for these alleles originate from the left humerus (DO 3994) of the same individual

## Conclusion

The study clearly demonstrates the possibility of presence/absence analyses of TEs in 3,000-year-old human remains from the Lichtenstein cave. These and earlier results indicate and prove the high quality of DNA preservation and the applicability of molecular analyses using the remains from this cave [44, 45, 52], but could not yet show the amplification of 500 bp fragments. Of 30 loci, we initially achieved 22 definite results (FAP in Table 1). With additional amplification using internal *Alu* primers, we could add four more results (*Alu*_3, *Alu*_14, *Alu*_19 and *Alu*_20), thus, 26 definite results (FAP and IAP in Table 1). The following *Alu* loci were incongruent with the family situation: *Alu*_2, *Alu*_7, *Alu*_16, *Alu*_26 and *Alu*_27. Amplification with internal *Alu* primers could place *Alu*_16, *Alu*_26 and *Alu*_27 in congruence with the family situation. The proposed verification technique is to check for possible presence bands by amplification with an internal *Alu* primer to get short target sequences of ~150 bp. Fragment lengths of more than 200 bp tend to be affected more often by allelic dropout events; therefore, short amplicons should be used. Thus, in further analyses, results that show only absence bands should be subjected to this strategy. Even less well-preserved DNA can be analyzed by this approach. The present study constitutes the basis for further investigations with more *Alu* loci and larger samples for microevolutionary studies in Central Europe. Such large-scale investigations would provide insight into *Alu* SINE-based microevolutionary processes in humans during the last few thousand years and help us comprehend the evolutionary dynamics of our genome. Current projects, like the 1,000 Human Genome Project, investigate human genetic variation and the interrelation of genotypes and phenotypes as well as variants in annotated genes and inherited genetic disorders [13, 53]. Through computational biology, the 1,000 Genome Project recently provided a genome-wide catalog of *Alu* polymorphisms for human populations [14]. A database with these group-specific insertions of polymorphic *Alu* elements is useful for future analyses with a larger dataset of Bronze Age Lichtenstein individuals – for instance, to investigate the geographic origin of Lichtenstein family members, who belong to the longest known family tree in the world. Through large-scale *Alu* element analyses of many individuals from the Bronze Age Lichtenstein cave, we may be able to detect human variability and evolution within one geographic region on a timeline. These data would represent a great supplement to recent human population genetic studies based on TEs.

## Methods

### Samples and DNA extraction

#### Samples

The skeletal material used for the present thesis originates from the Bronze Age Lichtenstein cave near Osterode in Lower Saxony, Germany. All bone material from the cave is stored at -20 °C in the Department of Historical Anthropology and Human Ecology of the Göttingen University, Lower Saxony, Germany. The DNA of the ancient individuals was extracted from three different members of a prehistoric family: father (left femur DO 1911), mother (left femur DO 3756) and daughter (left femur DO 3750). In all three cases, the DNA was extracted from the middle of the diaphysis. The modern DNA of the person from the United States of America was extracted from lymphocytes (CAU_1), and was provided with full written consent. This sample was ordered from “The Interstate Companies” (Memphis, Tennessee, USA) blood bank. The DNA of the modern positive control CAU_2 was extracted from cells of the buccal mucosa.

#### aDNA extraction from skeletal material with the QIAvac-24-plus

Fragments about 1 cm^2^ in size are sawed out of the middle of the diaphysis of the left femora. All outer surfaces of the fragments are removed to minimize the risk of a contamination with modern human DNA from, e.g., the excavation personal. The fragments are crushed with a steel mortar before they are powdered in a swing mill for 3 min at 24 swings per second. Afterwards, 0.25 g of the powder is transferred into a 15 ml FalconTube and 3900 μl of EDTA UltraPure™ 0.5 M pH 8 (Invitrogen™) and 100 μl of Proteinase K (600mAnson-U/ml) are added. This mixture is incubated for 18 h at 37 °C in a rotator. Now, an additional 50 μl of Proteinase K are added and the mixture is incubated at 56 °C for 2 h in a rotator. 50 μl of SDS (10 mg/ml) are added, followed by an incubation time of 5 min at 65 °C. The lysate is centrifuged at 3300rcf for 3 min to sediment surplus organic material. The lysate is transferred into a 50 ml FalconTube that contains 16 ml PB-Buffer (Qiagen) and 100 μl sodium acetate buffer (pH 5.2, 3 M, Sigma). After manually mixing the lysate, it is centrifuged at 3300rcf for 3 min. The DNA clean-up is conducted with minElute spin columns and funnels for large volumes using the QIAvac-24-plus (Qiagen). Deviating from the protocol, three wash steps with PE-buffer (Qiagen) are performed. The DNA is eluted in 60 μl RNase-free water (also cf. [54]).

#### Modern DNA

##### Blood sample

The DNA of the blood sample from CAU_1 is extracted with the Wizard Genomic DNA Purification Kit (Promega) following the producer’s protocol for extraction from whole blood samples (300 μl).

##### Buccal mucosa swab sample

A buccal mucosa swab from CAU_2 is transferred into a 2 ml reaction tube. 400 μl of G2-buffer (Qiagen) and 10 μl of Proteinase K are added, followed by incubation for 1 h at 56 °C and 350 rpm on an Eppendorf thermomixer comfort. Afterwards, 200 μl of the lysate is transferred into a clean tube and 1 ml of PB-buffer and 100 μl of sodium acetate buffer are added. After manually mixing the lysate, it is centrifuged at 3300rcf for 3 min. Now the DNA is cleaned up with minElute spin columns and large volume funnels as described above.

### *Alu* loci and primer design

*Alu* loci were chosen based on earlier publications with a population-genetics focus [23–26]. The site-specific *Alu* sequence was determined by using RepeatMasker [55]. An additional 500 bp flanking sequence on each site was extracted from the human reference genome (hg38) in NCBI [56]. The locus-specific primers were designed with PrimerSelect, version 10.1.2 (DNASTAR). The primer characteristics are a strong 5′ and a weak 3′ end by not exceeding a length of 30 bp; further, primer dimerization and hairpin formation were avoided to enhance the specificity and sensitivity of the reaction [38]. The total length of the target sequence (including the *Alu* insert) should be as short as possible, which usually resulted in amplicons of 450 bp to 500 bp (cf. also Additional file 4 for detailed information).

The internal *Alu* primers were designed based on a highly conserved region of the *Alu* sequence that is specific to the respective subfamily. Therefore, randomly selected *Alu* inserts of the respective *Alu*Y subfamily were aligned. The amplification always includes the *Alu* head.

A full list of the primer sequences is shown in the Additional file 5.

### PCR

All PCRs are conducted under the same conditions apart from the annealing temperatures. Depending on the energy profiles and melting temperatures of the primer sets and based on preliminary primer tests, different annealing temperatures, varying from 52 to 60 °C, are chosen. The amplification is conducted using the following cycling program: Initial hot start at 95 °C for 5 min; 40 cycles with denaturation at 94 °C for 1 min, annealing at 52 – 60 °C for 1 min, elongation at 72 °C for 1 min; a final soak at 10 °C for 10 min. The PCR is composed of 12.5 μl of Multiplex PCR Mastermix (Qiagen), 1 μl each forward and reverse primer, (both 20 μM working solution), 5 μl DNA for aDNA samples and 0.5 μl DNA (plus 4.5 μl RNase free water) for modern DNA samples and 5.5 μl of RNase-free water to get a final volume of 25 μl per reaction.

The amplification with an internal *Alu* primer was performed with an elongation time of 20 s. All other parameters are identical to the classical PCR approach.

For proof of authenticity, every DNA extract used in the study here presented was subjected to STR-typing by a multiplex amplification as described previously [57]. Deviating from this work, the sex discriminating amelogenin gene is arranged in the blue dye panel. The reaction mix is composed of 12.5 μl Multiplex PCR Mastermix (Qiagen), 2.85 μl multiplex primer mix, 4.65 μl RNase-free water and 5 μl DNA extract.

### Gel electrophoresis and fragment length estimation

Each amplification result is checked by ethidium bromide stained agarose gel electrophoresis (2.5 %). The fragment length determination is performed with a 50 bp molecular ladder (Invitrogen). For the electrophoresis, usually a voltage of 120 V and a run time of 30 min are applied.

The STR products are separated in a 50 cm capillary on an ABI 3500 Genetic Analyzer (Applied Biosystems) using POP-7™ Polymer for 3500/3500xL Genetic Analyzers and the 3500 Data collection Software (all Applied Biosystems). Allele determination is performed with GeneMapper Software 5 (Applied Biosystems).

### Cloning and sequencing

Cloning of PCR products is conducted with the Blue/White-Selection based pGEM®-T Easy Vector System (Promega). Deviating from the manufacturer’s protocol, 300 μl SOC medium (Invitrogen) is used to suspend the cells. Additionally, 50 μl – 100 μl of the cell suspension are plated. The Colony-PCR Mastermix is identical to the other PCRs except for the PCR primers. The primers pUC/M13 forward and reverse (Promega) are used in working concentrations of 20 μM. One colony replaces the DNA inset. The Colony PCR is conducted with the following program: Initial denaturation at 94 °C for 3 min; 30 cycles with denaturation at 94 °C for 30 s, annealing at 55 °C for 1 min, elongation at 72 °C for 50 s; final elongation at 72 °C for 2 min and final soak at 10 °C for 10 min. PCR products are purified with an isopropanol purification protocol: the PCR product is incubated with 83 μl HPLC water, 100 μl isopropanol (100 %) and 10 μl sodium acetate (3 M) for 10 min, then centrifuged at 13,200 rpm for 10 min in a conventional tabletop microcentrifuge. The supernatant is discarded and 150 μl of ethanol (70 %) is added. After another 10 min of centrifugation at 13,200 rpm, the supernatant is discarded, the pellet is dried and the desired amount of RNase-free water is added for resuspension. The sequencing reaction is composed of 4 μl Sequencing Buffer (5x), 2 μl BigDyeTerminator v1.1, 0.3 μl primer (20 μM), 6.7 μl HPLC water and 7 μl purified PCR product. Sequencing is performed in forward and reverse direction with the following program: Initial heating step at 94 °C for 3 min; 33 cycles with denaturation at 94 °C for 30 s, annealing at 55 °C for 1 min and elongation at 72 °C for 2.5 min; soak at 10 °C. Sequencing products are purified with NucleoSeq^®^ columns (Macherey-Nagel). The products are separated in a 50 cm capillary on an ABI 3500 Genetic Analyzer (Applied Biosystems) using POP-7™ Polymer for 3500/3500xL Genetic Analyzers and the 3500 Data collection Software (all Applied Biosystems). The sequences are edited in BioEdit version 7.2.5 [58] and submitted to a BLAST analysis. Finally, sequence data with the following accession numbers were deposited in GenBank: KU323383-KU323387.

### Kinship calculation

For kinship calculation (Reverse Parentage Index; RPI), the genotype probabilities are calculated: **RPI = X/Y**. The numerator (X) is the probability that a woman randomly selected from a population is type AB, that a man randomly selected from a population is type CD and that the child is type BC. The child gets one of the two alleles of the father and the mother, respectively. The probability that one allele of one parent is inherited by the child is 0.5. The denominator (Y) is the probability that a woman randomly selected from a population and unrelated to the child is type AB, that a man randomly selected from a population and unrelated to the child is type CD, and that a child randomly selected from a population is type BC (also cf. [59, 60]). The reverse parentage index for one STR system is calculated as follows:

$$ \mathrm{R}\mathrm{P}\mathrm{I}=\frac{\mathrm{X}}{\mathrm{Y}}=\frac{2{\mathrm{P}}_{\mathrm{A}}{\mathrm{P}}_{\mathrm{B}}\times 2{\mathrm{P}}_{\mathrm{C}}{\mathrm{P}}_{\mathrm{D}}\times 0.5\times 0.5}{2{\mathrm{P}}_{\mathrm{A}}{\mathrm{P}}_{\mathrm{B}}\times 2{\mathrm{P}}_{\mathrm{C}}{\mathrm{P}}_{\mathrm{D}}\times 2{\mathrm{P}}_{\mathrm{B}}{\mathrm{P}}_{\mathrm{C}}} $$

All single RPIs are multiplied to get a combined RPI.

## Additional files

Additional file 1: Genomic locations of all 30 *Alu* loci. This file contains precise locations of all 30 *Alu* loci investigated. (XLS 37 kb) Additional file 2: Sequences of randomly selected loci. This file contains sequencing results of 3 randomly selected loci with 5 sequences in total to check for authenticity. (PDF 18 kb) Additional file 3: List of all STR-typing results for the prehistoric individuals. The Microsoft Excel file contains four sheets with all STR-typing results for all prehistoric individuals investigated in this study. Sheets 1-3 contain the results of all 13 STR systems that were used for kinship calculation. The fourth sheet contains the STR-typing results that were obtained to check the authenticity of the DNA extracts. (XLSX 23 kb) Additional file 4: Detailed information on the *Alu* loci. The Microsoft Excel file contains detailed information about the exact lengths of the amplicons for every genomic *Alu* locus investigated in this study. Additionally shown are the lengths of the A-tails and the Target Site Duplications (TSD), as well as the sequences of the TSDs. (XLS 36 kb) Additional file 5: List of all *Alu* primers used. The Microsoft Excel file contains all primer sequences that were used in this study. (XLSX 10 kb)

## Abbreviations

aDNA: *ancient*DNA
LINEs: long interspersed elements
SINEs: short interspersed elements
TEs: transposable elements
