# Supplementary material for: Alu SINE analyses of 3,000-year-old human skeletal remains: a pilot study
Source: Mob DNA. 2016 Apr 18;7:7. doi: 10.1186/s13100-016-0063-y (PMC4836192; doi:10.1186/s13100-016-0063-y)
Supplement: Additional file 2: — Sequences of randomly selected loci. This file contains sequencing results of 3 randomly selected loci with 5 sequences in total to check for authenticity. (PDF 18 kb) [file 13100_2016_63_MOESM2_ESM.pdf]

DO 1911 Alu_1 (presence allele):

5' GGGGTTTGGATTGTGTATTATTTAGGGTAAGGAATATGTGCTGCTTTAGCAAATGAACTTGAAAATCTTTTATAGGGCCGGGCGCGGTGGCTCACGCCTGTAATCCCAGCACTTTGGGAGGCCGAGGCGGGCGGATCACGAGGTCAGGAGATCGAGACCATCCCGGCTAA 170

5' AACGGTGAAACCCCGTCTCTACTAAAAATACAAAAAATTAGCCGGGCGTAGTGGCGGGCGCCTGTAGTCCCAGCTACTTGGGAGGCTGAGGCAGGAGAATGGGGTGAACCCGGGAGGCGGAGCTTGCAGTGAGCCGAGATTGCGCCACTGCACTCCAGCCTGGGCGACAG 340

5' AGCGAGACTCCGTCTCAAAAAAAAAAAAAAAAGAAAAATCTTTTATAAAGTCTGGAGTCAGGACTGGC 408

DO 3750 Alu_1 (presence allele):

5' GGGGTTTGGATTGTGTATTATTTAGGGTAAGGAATATGTGCTGCTTTAGCAAATGAACTTGAAAATCTTTTATAGGGCCGGGCGCGGTGGCTCACGCCTGTAATCCCAGCACTTTGGGAGGCCGAGGCGGGCGGATCACGAGGTCAGGAGATCGAGACCATCCCGGCTAA 170

5' AACGGTGAAACCCCGTCTCTACTAAAAATACAAAAAATTAGCCGGGCGTAGTGGCGGGCGCCTGTAGTCCCAGCTACTTGGGAGGCTGAGGCAGGAGAATGGGGTGAACCCGGGAGGCGGAGCTTGCAGTGAGCCGAGATTGCGCCACTGCACTCCAGCCTGGGCGACAG 340

5' AGCGAGACTCCGTCTCAAAAAAAAAAAAAAAAGAAAATCTTTTATAAAGTCTGGAGTCAGGACTGGC 407

DO 3756 Alu_17 (presence allele):

5' TCTGTGTTCTTTGAATGTGTATTACTCTTaTCTGACAAATTAATAGACCTTTAAAAATTAAGGTCAGCCGGGCACGGTGGCTCACGCCTGTAATCCCAGCACTTTGGGAGGCCGAGGCGGGCGGATCACGAGGTCAGGAGATCGAGACCATCCCGGCTAAAATGGTGAAA 170

5' CCCCGTCTCTACTAAAAATACAAAAAATTAGCCGGGAGTAGTGGCGGGCGCCTGTAGTCCCAGCTACTTGGGAGGCTGAGGCAGGAGAATGGCGTGAACCCGGGAGGCGGAGCTTGCATGAGCCAAGATCCCGCCACTGCACTCCAGCCTGGGCGACAGAGCGAGACTCC 340

5' TCTCAAAAAAAAAAAAAAAAAAAAAAAATTAAGGTCTTCCTTCACTAATTTTTTTTAGTGCAGGAATGGTTTTTATAACATAGTGAATGTTAACAATTGAAAAGGAAAGTTACAGCTTTACATTC 465

DO 1911 Alu_22 (presence allele, amplification with internal Alu primer):

5' CGGTTGGKTTCAATGTCCTTTAAAAATAGCAGGATAATTTTTTTACSTGATTCTATAAATAAATCAAATTGAAGGCTGGGCGCGGTGGSTCACGCCKGTAATCCCAGCACTTTGGGAGGCCGAGGCGGGCGGATCACGGGGTCAGGAGATCGAGRCCATCCCGGC 165

DO 3750 Alu_22 (presence allele, amplification with internal Alu primer):

5' CGGTTGGTTTCAATGTCCTTTAAAAATAGCATGATAATTTTTTTACCTAATTCTATAAATAAATCAAATTGAAGGCTGGGCGCGGTGGCTCACGCCTGTAATCCCAGCACTTTGGGAGGCCGAGGCGGGCGGATCACGAGGTCAGGAGATCGAGACCATCCCGGC 165
